# Fructose and Non-Alcoholic Steatohepatitis

**DOI:** 10.3389/fphar.2021.634344

**Published:** 2021-02-08

**Authors:** Elke Roeb, Ralf Weiskirchen

**Affiliations:** ^1^ Department of Gastroenterology, Justus Liebig University Giessen and University Hospital Giessen, Giessen, Germany; ^2^ Institute of Molecular Pathobiochemistry, Experimental Gene Therapy and Clinical Chemistry (IFMPEGKC), RWTH University Hospital Aachen, Aachen, Germany

**Keywords:** non-alcoholic fatty liver, obesity, diabetes, lipogenesis, sugar, fructose, inflammation, fibrosis

## Abstract

**Background:** The excessive consumption of free sugars is mainly responsible for the high prevalence of obesity and metabolic syndrome in industrialized countries. More and more studies indicate that fructose is involved in the pathophysiology and also in the degree of disease of non-alcoholic fatty liver disease (NAFLD). In epidemiologic studies, energy-adjusted higher fructose consumption correlates with NAFLD in overweight adults. In addition to glucose, fructose, as an equivalent component of conventional household sugar, appears to have negative metabolic effects in particular due to its exclusive hepatic metabolism. Liver-related mortality is strictly associated with the degree of fibrosis, whereas the most common cause of death in patients suffering from NAFLD and non-alcoholic steatohepatitis (NASH) are still cardiovascular diseases. In this review article, we have summarized the current state of knowledge regarding a relationship between fructose consumption, liver fibrosis and life expectancy in NASH.

**Method:** Selective literature search in PubMed using the keywords ‘non-alcoholic fatty liver’, ‘fructose’, and ‘fibrosis’ was conducted.

**Results:** The rate of overweight and obesity is significantly higher in both, adult and pediatric NASH patients. The consumption of free sugars is currently three times the maximum recommended amount of 10% of the energy intake. The current literature shows weight gain, negative effects on fat and carbohydrate metabolism and NASH with hypercaloric intake of fructose.

**Conclusions:** Excessive fructose consumption is associated with negative health consequences. Whether this is due to an excess of energy or the particular metabolism of fructose remains open with the current study situation. The urgently needed reduction in sugar consumption could be achieved through a combination of binding nutritional policy measures including taxation of sugary soft drinks. Previous studies suggest that diet-related fructose intake exceeding the amount contained in vegetables and fruits lead to an increase of hepatic lipogenesis. Thus, further studies to clarify the protective contribution of low-fructose intake to positively influence NAFLD in industrial population are urgently required.

## Introduction

A positive energy balance and the consumption of free sugars are particularly the basis for the development of overweight and the metabolic syndrome. Fructose as a simple sugar occurs naturally in pome fruit (in apples and pears each about 6/100 g), berries (about 7.5/100 g), in exotic fruits (pomegranate and persimmon), and in honey (about 40/100 g) as well as in synthetic honey (US Food and Drug Administration, 2018). Table sugar normally is a double sugar, which is composed of one molecule each of glucose (grape sugar) and fructose. A significant proportion of the sugar intake nowadays comes from industrially manufactured foods that contain fructose-glucose syrup (i.e., high-fructose corn syrup). The term "free sugar" describes monosaccharides (glucose, fructose) and disaccharides (sucrose, table sugar) that either occur naturally in foods such as fruit and fruit juices or are added to industrially manufactured foods and beverages (US Food and Drug Administration, 2018).

The current WHO guideline recommends that free sugar should not be more than 10% of the total energy requirement of adults and children (Guideline, 2015). With an energy demand of 2000 kcal for an average adult, this would correspond to an amount of 50 g of sugar (17 sugar cubes ≈ 12 teaspoons of household sugar ≈ 500 ml of orange juice ≈ 5 oranges). A further reduction of free sugars to 5% of the estimated guide value of the total energy consumption to diminish the risk of dental caries is recommended by the WHO with limited informative value (Guideline, 2015; Schwingshackl et al., 2020). Children have a special role with regard to sugar intake, as they have an innate, evolutionarily advantageous preference for energy-dense and sweet foods (Rabenberg and Mensink, 2013). The uptake of sugar leads to an endogenous release of opioids, an effect that is used in pediatrics for painful procedures in newborns (Stevens et al., 2016; Gibbins and Stevens, 2001).

Both, prenatally and postnatally, exposure to certain flavors through amniotic fluid and breast milk has an influence on the development of taste preferences (Mennella et al., 2001; Nehring et al., 2015). In general, breast milk seems to have a positive effect on the diversity of food accepted by children due to the greater variety of flavors compared to infant formula (Fidler Mis et al., 2017). The individual sensitivity for sweets is also influenced by genetic polymorphisms of the taste receptors. The phenomenon of “flavor learning” in childhood was taken into account within the framework of the guideline of the ESPGHAN (European Society of Paediatric Gastroenterology, Hepatology and Nutrition), which recommends a maximum sugar intake of 5% of the overall energy requirement for children aged 2–18, corresponding to 16 g sugar (4 teaspoons) in a 4 year old boy. An even lower intake is recommended for children under 2 years of age, whose taste preference appears to be influenced more better (Fidler Mis et al., 2017). The actual proportion of free sugars in total energy intake is currently 13–14% for adults and 15–17.5% for children, and is thus still well above the maximum recommended amount (Schwingshackl et al., 2020; Bagus et al., 2016; Perrar et al., 2019). Sweets (34%) and fruit juices (22%) make up the main part of the intake of free sugars in childhood, but sugar-sweetened beverages also play a role, as they hardly lead to a feeling of satiety despite their high energy density contribute (Neuenschwander-Tetri, 2013). The intake of free sugars and sugary soft drinks correlates with a low socio-economic status (Rabenberg and Mensink 2013; Richter et al., 2012). The ‘German Health Interview and Examination Survey for Children and Adolescents‘ (KiGGS) study showed that children between 3 and 17 years of age consumed an average of two glasses (1 glass = 200 ml) of sugary drinks (juices, soft drinks, etc.) per day. Assuming a sugar content of around 10 g/100 ml of drink, this corresponds to around 40 g of free sugar or 160 kcal (Rabenberg and Mensink 2013; Ventura et al., 2011).

## Fructose and Non-alcoholic Fatty Liver Disease

**FIGURE 1:**
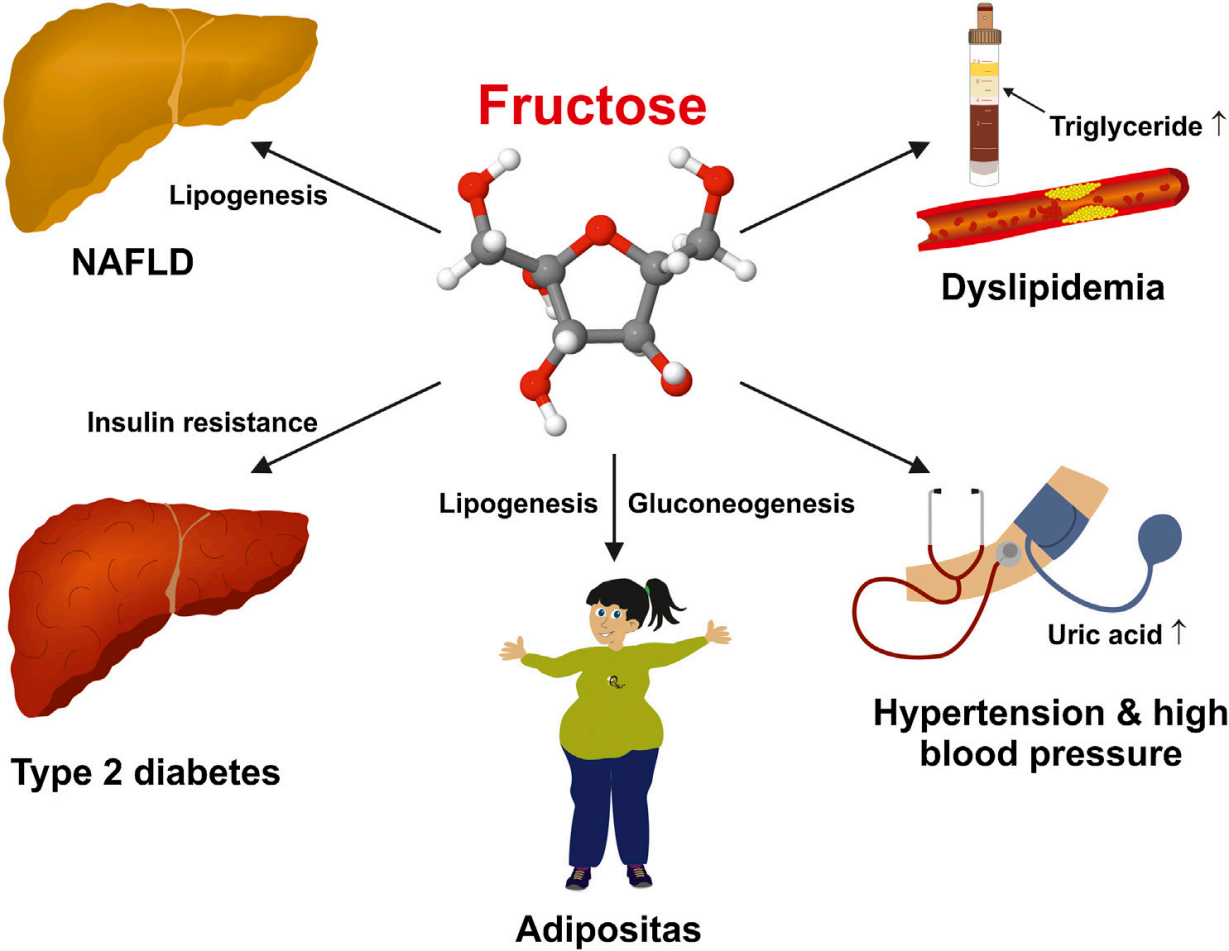
Deleterious effects of a high fructose intake on human health. Excessive fructose consumption is a risk factor for several chronic diseases including non-alcoholic fatty liver disease (NAFLD), obesity, dyslipidemia, insulin resistance/diabetes type 2, arterial hypertension, and hyperuricemia.

Fructose-rich diet forms can very quickly lead to almost all basic diseases of the metabolic syndrome. (Hannou et al., 2018). This medical condition is associated with trunk obesity, arterial hypertension, high serum sugar/impaired glucose tolerance (diabetes), elevated serum triglycerides, and reduced high-density lipoproteins (Figure 1).

**FIGURE 2:**
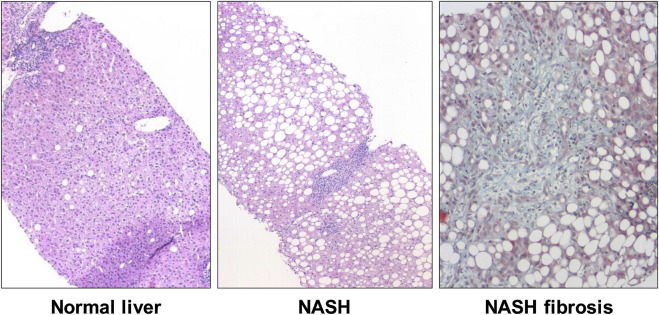
Histopathology of nonalcoholic steatohepatitis (NASH). Compared to normal liver **(left)**, histologic features of NASH include steatosis, ballooning, and lobular inflammation **(middle)**. Although not a requirement for the histological diagnosis NASH, fibrosis is often paired with the pathological changes **(right)**.

Due to its hepatic metabolism, fructose is suspected to be partly responsible for the development of non-alcoholic fatty liver disease (NAFLD). Most prandial fructose is not metabolized in the intestine but rather passes via the portal vein to the liver (Brown et al., 1997). The term NAFLD includes on the one hand the potentially reversible NAFL (non-alcoholic fatty liver), defined by a fat content of more than 5% of the hepatic parenchymal surface, as well as NASH (non-alcoholic steatohepatitis) with additional mixed-cell inflammatory infiltrates. NAFLD is etiologically closely linked to the metabolic syndrome and hyperalimentation and can lead to liver cirrhosis and/or liver cancer, especially hepatocellular carcinoma (HCC) (Roeb et al., 2015). The prevalence of NAFLD is estimated at around 26% in industrialized nations and has been characterized by a particularly significant increase in the number of cases in recent years. From 18.9% for men and 22.5% for women in 1998, the prevalence increased to 23.3% and 23.9% respectively by 2010 (Lammert et al., 2019). Fructose is the main nutrient responsible for the fatty degeneration of the liver, which can significantly lead to excess hepatic energy and, moreover, to fatty liver cells. Partly controversial retrospective data indicate higher fructose consumption in NAFLD patients and an influence on the extent of liver fibrosis (Figure 2) (Ouyang et al., 2008; Mosca et al., 2017; Abdelmalek et al., 2010).

Animal experiments have shown that diets enriched in fructose (70% of total energy intake) compared with a high glucose content show significant histological fatty liver disease and laboratory signs of liver cell damage (Jürgens et al., 2005; Kawasaki et al., 2009; de Castro et al., 2013). A prospective case-control study also found a connection between increased consumption of soft drinks and NAFLD (Abid et al., 2009). The enhanced intake of sugar and fructose is of great importance for the simple steatosis but also for the progression to severe forms of nonalcoholic fatty liver disease (NAFLD), such as nonalcoholic steatohepatitis (NASH), fibrosis (NASH fibrosis), and hepatocellular carcinoma (Lim et al., 2010). The aim of the present review is a compilation of the current data that support the importance of fructose intake for the development of liver fibrosis and life expectancy in NASH.

## Methods

A selective literature search in PubMed using the keywords ‘non-alcoholic fatty liver’, ‘fructose’, and ‘fibrosis’ was conducted at the beginning of December 2020. Using these search teams, a total of 135 publications were detected. After excluding pure *in vitro* trials and animal experiments, 35 publications were finally used for this review.

## Association of Fructose and NON-ALCOHOLIC FATTY LIVER DISEASE

Two meta-analyses dealt with the connection between fructose consumption and fatty liver disease. In 2014 Chiu et al. as well as Chung et al. were unable to demonstrate any effect of fructose on the intrahepatocellular fat content and the alanine aminotransferase level in studies in which fructose was used in an isocaloric exchange with other carbohydrates, while the hypercaloric intake of fructose negatively affects both parameters (Chiu et al., 2014; Chung et al., 2014). The findings of these studies suggest that the study situation is sometimes controversial. However, a high fructose consumption, which is accompanied by an excess of energy, seems to be associated with increased plasma glucose and triglyceride levels and significantly contributes to hepatic steatosis (Ter Horst and Serlie 2017). The extent to which fat as a macronutrient contributes to the development of NAFLD as well as the effects of high carbohydrate intake is the subject of current research.

**FIGURE 3:**
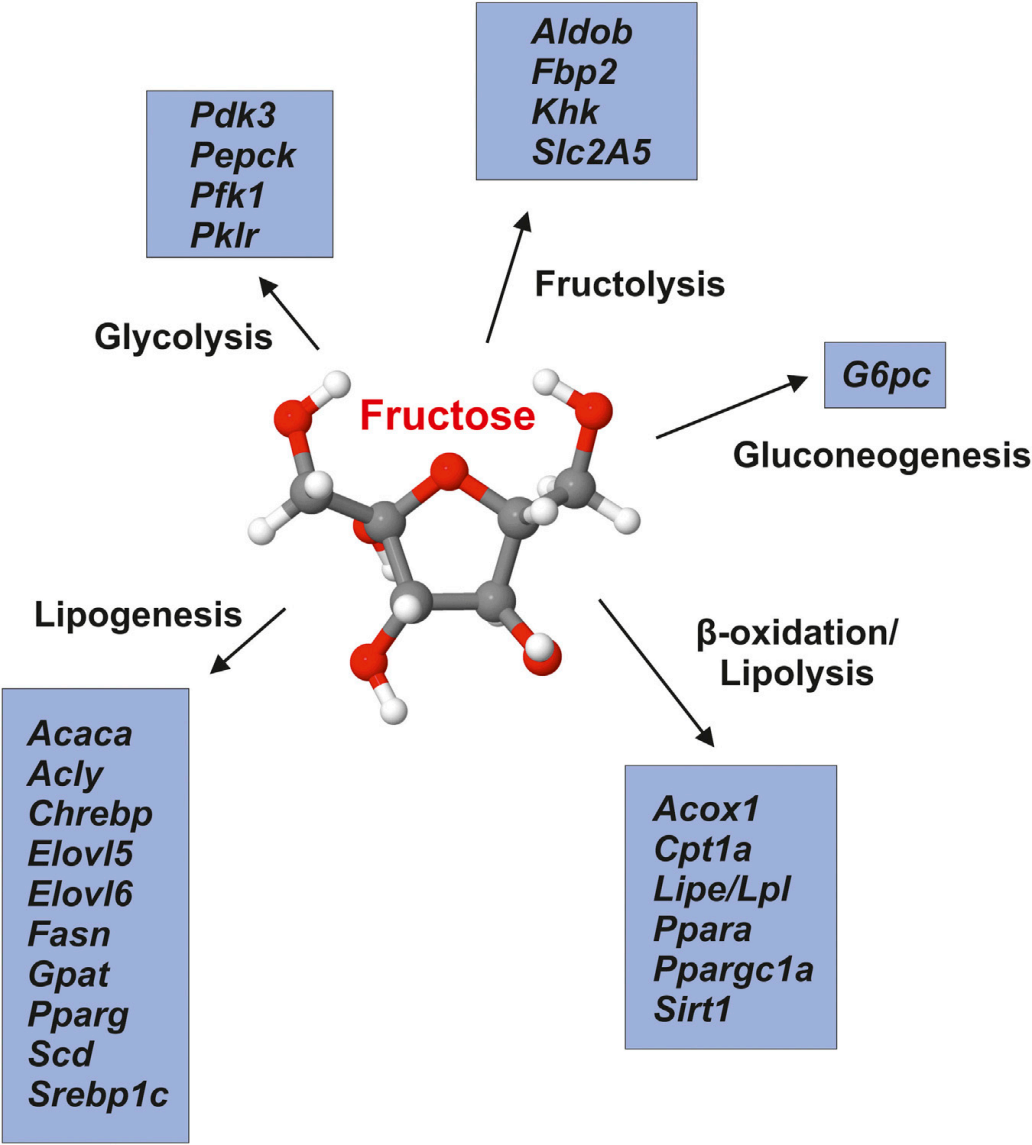
Fructose impacts hepatic energy metabolism. Fructose modulates the expression of a large number of genes that are involved in regulation of glycolysis, lipogenesis, *β*-oxidation/lipolysis, fructolysis and gluconeogenesis. Gene symbols (in alphabetical order) correspond to: *Acaca*, acetyl-CoA carboxylase-α; *Acly*, ATP citrate lyase; *Acox1*, acyl-coenzyme A oxidase one; *Aldob*, aldolase B; *Chrebp*, carbohydratre response element-binding protein; *Cpt1a*, carnitine palmitoyltransferase 1 (isoform a); *Elovl5*, elongation of very long chain fatty acids-like five; *Elovl6*, elongation of very long chain fatty acids-like six; *Fasn*, fatty acid synthase; *Fbp2*, fructose-1,6-bisphosphatase two; *G6pc*, glucose-6-phosphatase; *Gpat*, glycerol-3-phosphate acyltransferase (mitochondrial); *Khk*, ketohexokinase; *Lipe/Lpl*, lipase/lipoprotein lipase; *Pdk3*, pyruvate dehydrogenase kinase, isoenzyme three; *Pepck*, phosphoenolpyruvate carboxykinase one; *Pfk1*, phosphofructokinase (muscle type); *Pklr*, pyruvate kinase (liver and red blood cell); *Ppara*, peroxisome proliferation-activated receptor-α; *Pparg*, peroxisome proliferation-activated receptor-γ; *Ppargc1a*, peroxisome proliferation-activated receptor-γ coactivator 1-α; *Scd*, stearoyl-Coa desaturase; *Sirt1*, sirtuin one; *Slc2A5*, solute carrier family 2 (facilitated glucose/fructose transporter) member five; *Srebp1c*, sterol regulatory element-binding transcription factor 1 (isoform c). Adapted from (DiStefano 2020).

According to the experimental studies carried out so far, fructose acts as nutritional regulator impacting the expression of many genes engaged in fructolysis, *de novo* lipogenesis, gluconeogenesis, glycolysis, and lipolysis (e.g., fatty acid oxidation) by mechanisms associated with transcriptome analysis and epigenetic (Figure 3). We suspect that these effects of fructose intake are related to the respective nutritional status and gender of the test subjects. These effects persist in spite of fructose reduction, are passed on from mothers with high fructose consumption to their offspring and might influence the hepatic fat metabolism long before obesity and insulin resistance occur (DiStefano 2020). An important regulator of cholesterol metabolism showed decreased gene expression in males from fructose-fed mothers. In accordance Liver X-receptor gene promoter methylation was increased in males from fructose-fed mothers. Thus, maternal fructose intake produces a fetal programming that influences transcription epigenetically, and both hepatic mRNA gene expression and plasma parameters of cholesterol metabolism in adult progeny (Rodrigo et al., 2018).

The association between high fructose intake and obesity has led to analyze fructose and its impact on NAFLD. The consumption of sucrose and high fructose corn syrup rose exponentially about 1,000% during 1970 and 2010 and makes up to 10% of the calories in the daily diet (Abdelmalek et al., 2010). Of 60 ultrasound-proven NAFLD patients, 80% had an excessive soft drink intake compared to 17% of healthy controls. In this same study, NAFLD subjects consumed five times more carbohydrates from soft drinks compared to controls (Abid et al., 2009). Thus, regular soft drink consumption seems to be a predictor of NAFLD and the metabolic syndrome with hypertriglyceridemia, pathological glucose tolerance in liver, weight gain, and insulin resistance (Nseir et al., 2010). High fructose concentrations were found in dates, honey, raisins, and sweet cherries as well as in cola (see Table 1).

**TABLE 1:** Fructose in different foods.

Food	Glucose	Fructose
	Grams per 100 g of food	Grams per 100 g of food
Apple	3.4	6.9
Artichoke	0.8	1.7
Banana	7.4	7.2
Broccoli	0.7	1.1
Butter	0.0	0.0
Carrot	2.5	2.4
Cashew	3.0	2.9
Cauliflower	1.0	0.9
Chard	0.3	0.4
Chicken meat	0.0	0.0
Chicory	1.5	0.9
Cola	5.0	5.0
Corn	3.1	2.9
Cranberry	3.3	0.7
Cucumber	0.8	0.9
Currant red	3.5	3.8
**Dates**	**34.0**	**31.0**
Egg yolk	0.2	0.0
Fennel	1.5	1.3
figs	7.2	5.9
Grapes	7.3	8.2
Herring	0.0	0.0
**Honey**	**35.2**	**40.2**
Honeydew melon	3.9	4.2
Kale	1.2	1.4
Kiwi	5.1	5.2
Kohlrabi	1.9	1.8
lamb's lettuce	0.5	0.3
Leek	1.7	1.5
Lemon	1.2	1.1
Lentils	0.7	1.0
Mango	5.5	8.2
Milk	0.0	0.0
Oats	0.4	0.4
Orange	4.3	4.4
Papaya	2.9	3.0
Paprika red	2.7	3.0
Pasta	0.1	0.1
Peach	4.1	4.0
Peanuts	1.3	1.3
Pear	2.8	7.2
Peas	3.1	3.1
Pineapple	4.7	5.1
Plum	3.9	5.2
Pomegranate	7.9	8.5
Potato	0.5	0.4
Protein	0.4	0.0
Pumpkin	1.1	0.8
**Raisins**	**31.2**	**31.6**
Raspberry	2.5	3.1
Rice	0.1	0.1
Salad	0.5	0.6
Salmon	0.0	0.0
Savoy	2.0	1.8
Sunflower seeds	1.4	1.4
Sweet cherry	13	12
Tomatoes	1.2	1.5
Trout	0.0	0.0

Indicated is the total fructose and glucose content, which always includes free fructose and half the sucrose value. The values in this table are calculated from mean values according to (Souci et al., 2016).

In adults with NAFLD, daily fructose ingestion was associated with reduced hepatic steatosis but increased fibrosis (Abdelmalek et al., 2010). In addition, Abdelmalek et al. stated that in older patients, e.g., in adults age > or = 48 years, a daily fructose consumption was associated with histologically proven significant increased hepatic inflammation and hepatocyte ballooning. Even in healthy adult men high-fructose intake was associated with increased DNL and liver fat in healthy men fed weight-maintaining diets (Schwarz et al., 2015).

## The Way How Fructose Influences NON-ALCOHOLIC FATTY LIVER DISEASE

**FIGURE 4:**
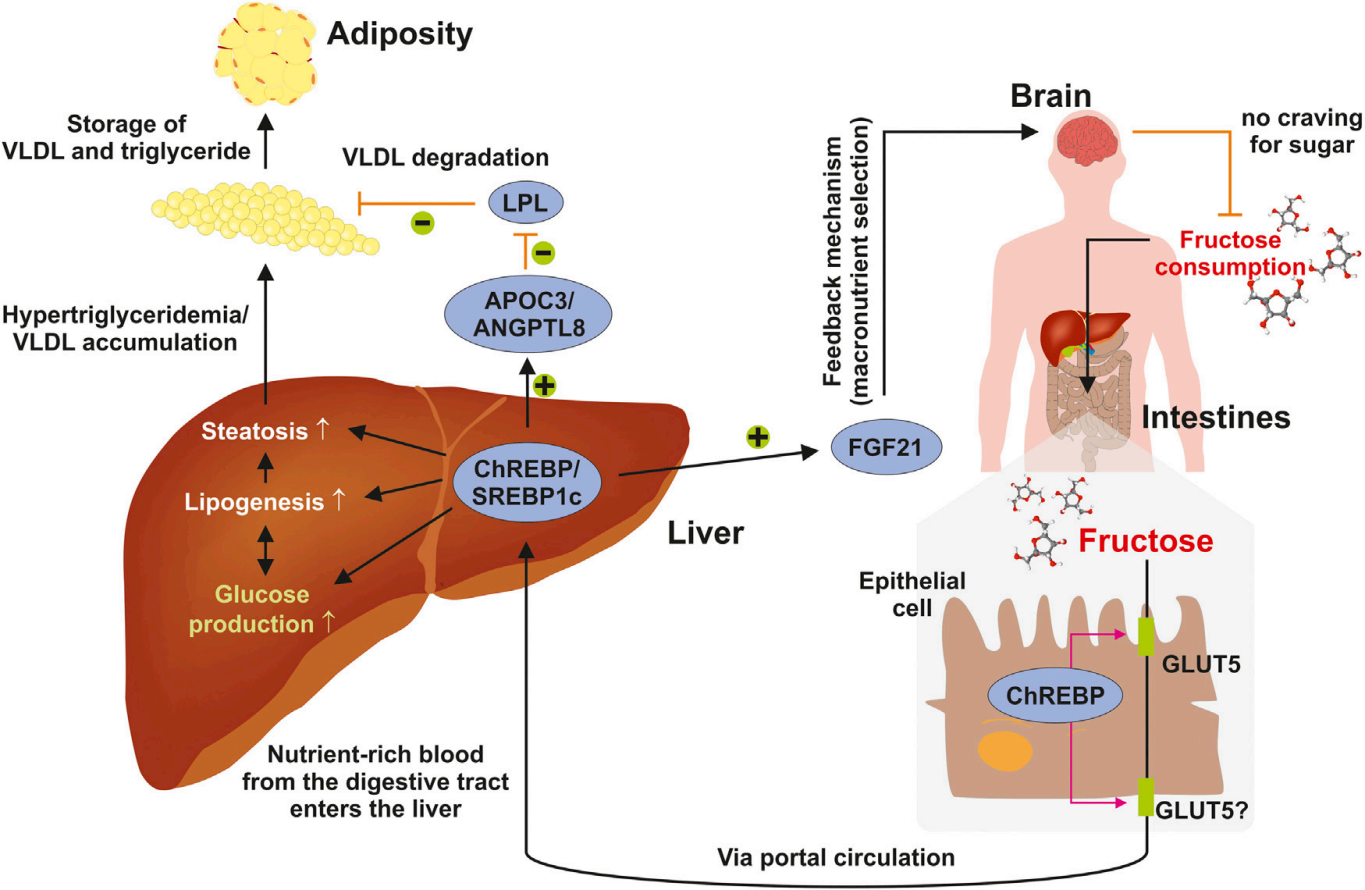
Elevated fructose intake trigger fatty liver disease. Consumed sugar such as fructose moves from the intestinal lumen to the blood circulation through a facilitated passive transport across the apical border of enterocytes using the facilitative glucose/fructose transporter GLUT5. It is then transported into the liver through the hepatic portal vein. In the liver fructose is metabolized to glucose and drives lipid synthesis resulting in steatosis, elevated concentrations of triglycerides, and adiposity. Under physiological conditions, these processes are tightly regulated finely adjusted in crosstalk with the brain, which integrates signals impacting food intake, energy expenditure, insulin secretion, hepatic glucose production, and fatty acid metabolism. For details see text. Abbreviations used are: APOC3, apolipoprotein C-III; ANGPTL8, angiopoietin-like eight; FGF21, fibroblast growth factor 21; VLDL, very low density lipoprotein. Adapted from (Hannou et al., 2018).

The individual diet is highly relevant for the development of NAFLD and both, risky (e.g., fructose) and protective eatables (Mediterranean diet), have been identified, but the contribution of exorbitant calories remains crucial (Marchesini et al., 2016). Fructose metabolism in the liver is not a tightly regulated biochemical pathway in contrast to glycolysis, where the rate-limiting enzyme phosphofructokinase, is present (Nd 2019). Fructose consumption enables disturbances of metabolic pathways that might result in excessive hepatic fat accumulation. As a central organ involved in regulating energy homeostasis, the liver coordinates interactions with other organs that are critical for the maintenance of energy homeostasis. Dietary sugars such as fructose move from the small intestine into the blood circulation by means of a passive transport process throughout the apical border of enterocytes that is triggered by members of the facilitative glucose/fructose transporter (GLUT) family such as GLUT5 (Merino et al., 2019) (Figure 4). Thereafter, the sugar is transported toward the liver via the hepatic portal vein where it activates the production of glucose and lipid synthesis. Increased hepatic lipogenesis results in steatosis and elevated concentrations of triglycerides that on long-term give cause to adiposity. Under physiological conditions, these processes are tightly regulated. Chronic fructose consumption leads to activation of the sterol regulatory element-binding protein 1c (SREBP1c) and the carbohydrate-responsive element-binding protein (ChREBP) that in turn provokes the expression of the liver-derived hormone fibroblast growth factor 21 (FGF21) regulating energy homeostasis and protecting the liver from fructose-induced metabolic disease (Hannou et al., 2018). Moreover, ChREBP transactivates expression of the apolipoprotein C-III (APOC3) and angiopoietin-like 8 (ANGPTL8), which both lower the activation of lipoprotein lipase (LPL) and limit the clearance of very low density lipoproteins (VLDL) (Hannou et al., 2018). The metabolic activity of the liver is further modulated in crosstalk with the brain, which modulates different aspects of metabolism, including food ingestion, energy consumption, insulin secretion, glucose metabolism, and fatty acid production in adipose tissue (Roh et al., 2016).

Taken together fructose seems to functions as both, as a substrate and as an inducer of hepatic *de novo* lipogenesis (DNL) (Jegatheesan and De Bandt, 2017). Fructose absorbed from the portal blood into the liver cells is converted to fructose-1-phosphate by fructokinase and thereby activated for further metabolic steps. Fructose-1-phosphate is split into d-glyceraldehyde and dihydroxyacetone phosphate by the liver-specific fructose-1,6-diphosphate aldolase. The latter can be broken down further via the corresponding reaction of glycolysis or used for gluconeogenesis. Thus, fructose can be broken down into pyruvate more quickly than glucose since the rate-limiting reactions of glycolysis (glucokinase and phosphofructokinase reaction) are bypassed (Jegatheesan and De Bandt, 2017). By saturating the glycolytic pathway, high fructose intake might result in an accumulation of glycolysis intermediates which can be converted to glycerol-3-phosphate used in triglyceride (TG) synthesis. Excessive consumption of fructose, however, might additionally induce the deterioration of the intestinal barrier and induce inflammation (Kawabata et al., 2019). Very recently it was shown that fructose-elicited endotoxaemia activates Toll-like receptor (TLR) signaling in liver macrophages, a process that could be blocked experimentally. To prove that hypothesis in a murine model, the restoration of barrier function was associated with reduced DNL and reduced hepatosteatosis, attenuated HCC formation and minor expression of lipogenic and inflammatory genes. In addition, an antimicrobial peptide prevented fructose-induced NAFLD (Todoric et al., 2020).

Also Lambertz et al. reviewed that fructose ingestion provokes a transformation of the gut microbiome, leading to leaky gut with enhanced permeability of the intestinal barrier, hepatic inflammation, increasing insulin resistance and - last but not least - liver fibrosis (Lambertz et al., 2017). In addition, they discuss fructose-associated changes of tight junction proteins affecting the intestinal permeability, thus resulting in an entry of bacteria and bacterial endotoxins into the blood stream. A cross-sectional epidemiological study has associated fructose uptake to the degree of hepatic fibrosis, eg fibrosis grade, in fatty liver disease. Clinical trials revealed that ingestion of fructose-containing beverages, with either fructose or sucrose, contribute to the development of NAFLD in comparison to isocaloric alternative soft drinks. In addition genetic polymorphisms increasing the uptake of glucose into lipogenic pathways are associated with NAFLD (Neuschwander-Tetri 2013). Moreover, Silbernagel et al. detected a correlation of visceral and liver fat content with cholesterol synthesis even in wholesome humans. In addition, they showed that cholesterol synthesis seems to depend on the uptake of fructose and glucose. (Silbernagel et al., 2012). However, prospective multicenter studies analyzing whether marked transformations of hepatic fat amount will influence cholesterol homeostasis are still missing.

Fructose intake increased hepatic *de novo* lipogenesis, and lipoprotein lipase activity was downregulated postprandial in humans consuming fructose in comparison with subjects consuming glucose. The authors conclude that increased *de novo* lipogenesis as well as decreased lipoprotein lipase-mediated clearance are part of the fructose-induced postprandial hypertriglyceridemia (Stanhope et al., 2009). Some years later, fructose was proposed as a key player in the formation of NAFLD (Basaranoglu et al., 2013). High-fructose corn syrup in soft drinks and other carbohydrate-sweetened beverages is a blend that is typically composed of 55% fructose, 41% glucose, and 4% complex polysaccharides. A higher intake of these beverages meaning enhanced fructose consumption has been associated with obesity, type 2 diabetes, and NAFLD in the USA. Thus, fructose provokes hepatic stress, phosphorylation of JNK and finally reduced hepatic insulin signaling (Basaranoglu et al., 2013). Moreover, it is indisputable that fructose decreases insulin sensitivity, and increases visceral adiposity in overweight/obese adults. But how does it work in lean subjects with NAFLD?

## ‘LEAN NAFLD’ Subjects

The term ‘lean NAFLD’ quotes to liver steatosis in slim patients or patients of normal weight, according to the per region-specific body mass index (BMI). Similar to the pathogenesis of NAFLD in obese persons, fructose consumption may also be important in the development of NAFLD in leans (Kumar and Mohan, 2017). Soft drink consumption for example seems to be associated with NAFLD in the absence of conventional risk factors (Assy et al., 2008). Independent of the diagnosis metabolic syndrome, NAFLD patients displayed higher soft drink consumption in a study published by Abdi and coworkers (Abid et al., 2009). In particular the study revealed that the NAFLD patients ingested five times as much carbohydrates in soft drinks in comparison to healthy controls (40% vs. 8%, *p* < 0.001). About 7% of patients just drank one soft drink daily, over 50% consumed two to three soft drinks per day, and 38% had more than four soft drinks per day for most days within a half year. The most popular drink was Coca-Cola (regular: 32%; diet: 21%), the second most popular fruit juices (47%) (Abid et al., 2009). Table 1 gives an overview of the fructose content of various fruits and fruit juices.

On the other hand, patients with fructose 1-phosphate aldolase B deficiency characterized by hereditary fructose intolerance also exhibit increased intrahepatic triglyceride accumulation suggesting that both the increasing concentration of fructose-1-phosphate and the derogation of *β*-oxidation seem to be involved in NAFLD pathogenesis. In patients with this inborn error, a higher intrahepatic triglyceride concentration with regard to controls was associated with impaired glucose tolerance. The accumulation of intermediates of fructolysis might cause intrahepatic triglyceride accumulation via impaired *β*-oxidation (Simons et al., 2019).

## No Hints for Negative Effects of Fructose

We don't want to conceal that there are other statements to fructose consumption in men. Three review articles argue indeed against any negative effects of fructose on human health (Tappy and Lê 2010; Rippe et al., 2017; White 2013). In addition, there is no concrete evidence that fructose consumption in normal ranges has serious consequences (Tappy and Lê 2012). Whether ingestion of smaller amounts of fructose over longer time periods stimulates *de novo* lipogenesis or increases intrahepatic fat concentration has not been circumstantiated in epidemiological studies and has to be assessed (Tappy and Lê 2012).

The ingestion of fruits and vegetables seems to represent a protection for various diseases, such as type 2 diabetes. In addition fruits and vegetables were correlated with a lower risk of overall and cardiovascular disease (Wang et al., 2016). In an interventional clinical trial in order to unravel the pathogenetic mechanisms of fructose in comparison to glucose consumption Smajis et al. demonstrated that the consumption of a high dose of fructose over 8 weeks had no influence on important metabolic consequences in case of a stable energy intake, slightly lower body weight, and potentially incomplete absorption of the ingested fructose (Smajis et al., 2020). Thus, young and healthy humans might at least for a short time period be able to compensate a higher fructose intake.

## Lipogenic Markers and Triacylglycerol Synthesis

Liver fibrosis induced by a high fructose intake was associated with increased body weight, hunger-satiety system dysregulation, increased insulin concentration, dysregulated lipid metabolism, lipoperoxidation and inflammation. In addition, enhanced levels of hepatic glucose-6-phosphate dehydrogenase (G6PD) and malic enzyme activity, the NAD(*p*)H/NAD(*p*)^+^ ratios, the reduced glutathione concentration and increased expression of lipogenic and fibrotic markers were described. All these changes were reduced by the application of nicotinamide (Loza-Medrano et al., 2020). Specifically, nicotinamide reduced the activity and expression of G6PD and malic enzyme. This finding was associated with a reduction of the NADPH/NADP^+^ ratios, rising GSH levels and decreased lipoperoxidation and inflammation, improving fibrosis and NASH development. The manipulation of NADPH-producing enzymes was attended by the antifibrotic, antioxidant and antilipemic effects of nicotinamide (Loza-Medrano et al., 2020).

Diacylglycerol acyltransferase (DGAT)1 and DGAT2, catalyze the final step of triglyceride synthesis (Harris et al., 2011). A recent study indicates that hepatic DGAT2 deficiency leads to a reduction of diet-induced hepatic steatosis, thus supporting the application of DGAT2 inhibitors as a therapeutic approach to ameliorate NAFLD and associated diseases (Gluchowski et al., 2019). Based on these findings, the inhibition of DGAT2 is regarded as a promising therapeutic approach for NAFLD/NASH in humans.

During cholestasis, the expression of genes encoding proteins involved in triacylglycerol (TAG) synthesis and *de novo* lipogenesis (*AGPAT1*, *GPAT1*, *MGAT1*, *DGAT1*, *DGAT2*, *FASN*, *HMGCS*1, *ACC1*, *SREBP1c*, and *PPARγ*) was downregulated (Irungbam et al., 2020). The reduced expression of *AGPAT1*, *GPAT1*, *MGAT1*, and *DGAT2* implicated that FFAs cannot be utilized for TAG synthesis. But increased free fatty acid (FFA) levels in parallel with reduction a of TAG synthesis and accumulation along with increased lipolysis during cholestasis seems to facilitate the acceleration of liver injury (Zahner et al., 2017).

## Intervention and Therapeutic Approaches in Non-alcoholic Fatty Liver Disease

Presently, there is no approved pharmacologic therapy to universally treat NAFLD. However, intensive lifestyle modifications such as drinking of unsweetened coffee and avoiding fructose corn syrup are reasonable specific dietary recommendation for patients suffering from NAFLD (Malhotra and Beaton 2015). Mediterranean diets high in vegetables, fish, nuts, grains, fruits, and olive oil enriched with unsaturated fats have been shown to beneficially effect or negatively correlate with NAFLD. In contrast, consumption of a typical Western diet, which includes soft drinks, fructose, red meat and saturated fatty acids predispose for NAFLD development (Zelber-Sagi et al., 2017). Short-term interventions by macronutrient manipulations might influence hepatic steatosis illustrating both pro- and anti-steatitic effects. In line, macronutrient manipulations that restrict uptake of carbohydrates and saturated fatty acids are reported to have efficacious effects (Stokes et al., 2019). A detailed literature review focusing on established principal has developed five key dietary recommendations including 1) traditional dietary patterns such as the Mediterranean diet, 2) limiting of excess fructose consumption and processed foods and beverages enriched in fructose, 3) replacement of dietary saturated fatty acids by long-chain omega-3 rich foods and monounsaturated fatty acids, 4) substitution of processed food, fast food, commercial bakery goods, and sweets by unprocessed or minimally processed foods that are high in fiber, including whole grains, vegetables, fruits, legumes, nuts, and seeds, and finally 5) reduction of excess alcohol consumption (George et al., 2018). Consequently, NAFLD incidence and progression is significantly reduced by improving diet quality following these guidelines. It is supposed that these benefits result from the collective effect of dietary patterns rather than from one single measure (George et al., 2018).

There is also clear evidence that regular exercise is beneficial to reduce the overall hepatic lipid content. In particular, diverse studies have shown that vigorous-intensity exercise is inversely correlated with NAFLD severity (Schon and Weiskirchen, 2016). This was particularly demonstrated in a study that enrolled more than 5,700 participants and found a dose-response relationship between physical activity and the occurrence of NAFLD (Kalathiya et al., 2015). Moreover, some clinical studies have demonstrated that short-term exercise, when conducted regularly, is already sufficient to reduce hepatocyte apoptosis in obese patients suffering from NAFLD by improving insulin sensitivity and restoration the general oxidative capacity, suggesting that active lifestyle and caloric restriction are beneficial in decreasing histological signs of fatty liver disease (Schon and Weiskirchen, 2016).

## Conclusion

NAFLD representing a form of chronic hepatitis is one of the most important and rising causes of liver disease worldwide. Respective patients have a high frequency of metabolic comorbidities such as obesity, impaired insulin resistance and dyslipidemia. Presently, there are only limited treatment options. The current standard of care for NAFLD patients is the limitation and control of risk factors and the advice to follow recommendations for lifestyle changes. In particular, weight loss provoked by dietary restriction and regular physical exercise can be beneficial in regress of liver disease. A Mediterranean diet can significantly reduce liver fat mass even without weight loss and is most recommended. However, there are also a substantial proportion of lean patients with NAFLD. The consumption of unsweetened coffee, reduction in fructose intake, and the avoidance of foods enriched in saturated fats are reasonable specific dietary recommendations for NAFLD and NASH patients. Sedentary activity patterns contribute as well as ingestion of low amounts of polyunsaturated fatty acid to NAFLD and metabolic syndrome. But in the case of a diverse diet, it is very difficult to emphasize the importance of one single nutrient. Thus, high-quality basic research and well-defined randomized clinical trials assessing dietary interventions that focus on liver-specific endpoints-are urgently needed as a priority.
